# Augmented Reality in Vascular and Endovascular Surgery: Scoping Review

**DOI:** 10.2196/34501

**Published:** 2022-09-23

**Authors:** Joshua Eves, Abhilash Sudarsanam, Joseph Shalhoub, Dimitri Amiras

**Affiliations:** 1 Imperial Vascular Unit Imperial College Healthcare NHS Trust London United Kingdom; 2 Department of Surgery & Cancer Imperial College London London United Kingdom; 3 Department of Radiology Imperial College Healthcare NHS Trust London United Kingdom

**Keywords:** augmented reality, surgery, vascular, endovascular, head-mounted display, mobile phone

## Abstract

**Background:**

Technological advances have transformed vascular intervention in recent decades. In particular, improvements in imaging and data processing have allowed for the development of increasingly complex endovascular and hybrid interventions. Augmented reality (AR) is a subject of growing interest in surgery, with the potential to improve clinicians’ understanding of 3D anatomy and aid in the processing of real-time information. This study hopes to elucidate the potential impact of AR technology in the rapidly evolving fields of vascular and endovascular surgery.

**Objective:**

The aim of this review is to summarize the fundamental concepts of AR technologies and conduct a scoping review of the impact of AR and mixed reality in vascular and endovascular surgery.

**Methods:**

A systematic search of MEDLINE, Scopus, and Embase was performed in accordance with the PRISMA (Preferred Reporting Items for Systematic Reviews and Meta-Analyses) guidelines. All studies written in English from inception until January 8, 2021, were included in the search. Combinations of the following keywords were used in the systematic search string: (“augmented reality” OR “hololens” OR “image overlay” OR “daqri” OR “magic leap” OR “immersive reality” OR “extended reality” OR “mixed reality” OR “head mounted display”) AND (“vascular surgery” OR “endovascular”)*.* Studies were selected through a blinded process between 2 investigators (JE and AS) and assessed using data quality tools.

**Results:**

AR technologies have had a number of applications in vascular and endovascular surgery. Most studies (22/32, 69%) used 3D imaging of computed tomography angiogram–derived images of vascular anatomy to augment clinicians’ anatomical understanding during procedures. A wide range of AR technologies were used, with *heads up* fusion imaging and AR head-mounted displays being the most commonly applied clinically. AR applications included guiding open, robotic, and endovascular surgery while minimizing dissection, improving procedural times, and reducing radiation and contrast exposure.

**Conclusions:**

AR has shown promising developments in the field of vascular and endovascular surgery, with potential benefits to surgeons and patients alike. These include reductions in patient risk and operating times as well as in contrast and radiation exposure for radiological interventions. Further technological advances are required to overcome current limitations, including processing capacity and vascular deformation by instrumentation.

## Introduction

### Rationale

Emerging technologies are transforming vascular surgery at a rapid pace. In particular, the introduction of endovascular techniques has opened the way for a Cambrian explosion of technological evolution in terms of both hardware [1] and software [2].

One such technology is augmented reality (AR), which aims to enhance clinicians’ ability by offering intuitive augmentation of the real environment with computer-generated real-time input of virtual information. AR lies on the continuum from virtual reality (VR), in which the user is immersed in a completely virtual setting, to real life. AR allows for minimal interaction between the virtual and real worlds, whereas mixed reality (MR) involves a combination of the real and virtual worlds where both elements are able to interact [3].

Complications in vascular surgery such as wound infections are associated with the extent of dissection, size of the wound, and duration of the surgery [4]. Anatomical localization with AR may help in reducing complications and improving overall outcomes. Although the “Getting It Right First Time” program used a principle of standardization across vascular surgery to improve outcomes in the United Kingdom [5], AR has promise in allowing for personalization taking into account individual patient anatomy and pathology while standardizing the technical approach by providing intraprocedural guidance.

Surgical AR uses a range of technologies, including everyday smartphone devices [6] and commercial products, including AR head-mounted displays (HMDs), which offer developers flexibility, allowing clinicians to experience virtual content that is overlaid directly onto the present reality [3].

Across a number of surgical specialties and subspecialties, the potential of AR as an important training tool has been identified [7]. With increasingly specialized surgical practice, more patients with comorbidities, and prevalent ethical challenges for surgical training, trainees have become increasingly reliant on simulation. Simulation has been demonstrated to effectively reduce training risks and costs [8]. Both VR and AR have been applied to surgical simulation successfully [9]; however, the superimposition of real-time information onto the real world and the flexibility offered by AR could make it a more realistic and adaptable simulation tool [10].

Literature on the applicability of AR in vascular surgery is extremely limited [11]. A recent review on the applicability of HMDs and smart glasses in vascular surgery highlighted the application of AR HMDs in a number of surgical specialties. However, only 4 papers in this review reported applications relevant to vascular surgery in particular [11]. To date, no review has been conducted on the application of the spectrum of AR technologies in the fields of vascular and endovascular surgery.

### Objectives

This scoping review aimed to systematically search the literature, identify the current applications of AR in vascular and endovascular surgery, and summarize key results and learning points while identifying avenues and gaps for future research in this evolving field.

## Methods

### Protocol

A scoping review was chosen after advice from PROSPERO (international prospective register of systematic reviews), and the PRISMA-ScR (Preferred Reporting Items for Systematic Reviews and Meta-Analyses extension for Scoping Reviews) [12] was used to ensure validity.

### Eligibility Criteria

The inclusion criteria were (1) studies in the English language, (2) a minimum of level V evidence using the Oxford Centre for Evidence-Based Medicine 2011 Levels of Evidence, (3) use of AR in vascular or endovascular surgery, and (4) applicability to clinical practice or training reported.

The exclusion criteria were (1) review articles or conference abstracts, (2) non–English-language articles, (3) articles lacking an available full text, (4) use of AR outside of vascular or endovascular surgery, and (5) use only of VR.

### Search and Information Sources

A systematic search of the MEDLINE, Scopus, and Embase databases was performed, allowing for access to a range of global clinical, scientific, and engineering research. All studies written in English from inception until January 8, 2021, were included in the search.

The following keywords were used in the systematic search string for all 3 database searches: (“augmented reality” OR “hololens” OR “image overlay” OR “daqri” OR “magic leap” OR “immersive reality” OR “extended reality” OR “mixed reality” OR “head mounted display”) AND (“vascular surgery” OR “endovascular”). No limits were applied.

### Selection of Sources of Evidence

First, a blinded and independent process of selection based on titles and abstracts was performed without collusion by 2 authors (AS and JE), with a third author (JS) consulted with regard to discrepancies. Next, a selection of eligible studies was conducted by analyzing the full texts.

### Data Items

Each study was evaluated individually, and the following variables were sought for data collection: year of study, category (endovascular surgery [aortic, peripheral, venous, or visceral], open surgery, and training), applied study or concept and design, number of patients (if the study was clinically applied), risk of bias (see the following section for the tools used), characteristics of the study group and operators, methods and outcomes, imaging type, and type of display (if applicable).

### Critical Appraisal of Individual Sources of Evidence

The quality of the data was evaluated using the Cochrane tools Risk of Bias 2 for randomized trials and Risk of Bias In Non-randomized Studies of Interventions for nonrandomized trials [13,14]. For those studies that included human participants but did not meet the criteria for the aforementioned scoring tools, the Johanna Briggs Institute Critical Appraisal Checklist was used, with a score derived based on the checklist. A score of 1 was assigned for *Yes*, a score of 0.5 was assigned for *unclear*, and a score of 0 was assigned for *no*. The results were presented as percentages. A higher percentage represented a reduced risk of bias.

### Data Charting Process and Synthesis

Data were extracted from eligible studies into evidence tables to summarize the following: year of publication, type of study design, number of patients, method, and outcome. A second table presents more technical aspects of the research, including the type of imaging, methods for tracking or registration, and display. The data collected in the evidence tables were used to define the main themes of discussion. Any data related to the application of AR in vascular and endovascular surgery could be synthesized.

## Results

### Selection of Sources of Evidence

**Figure 1:**
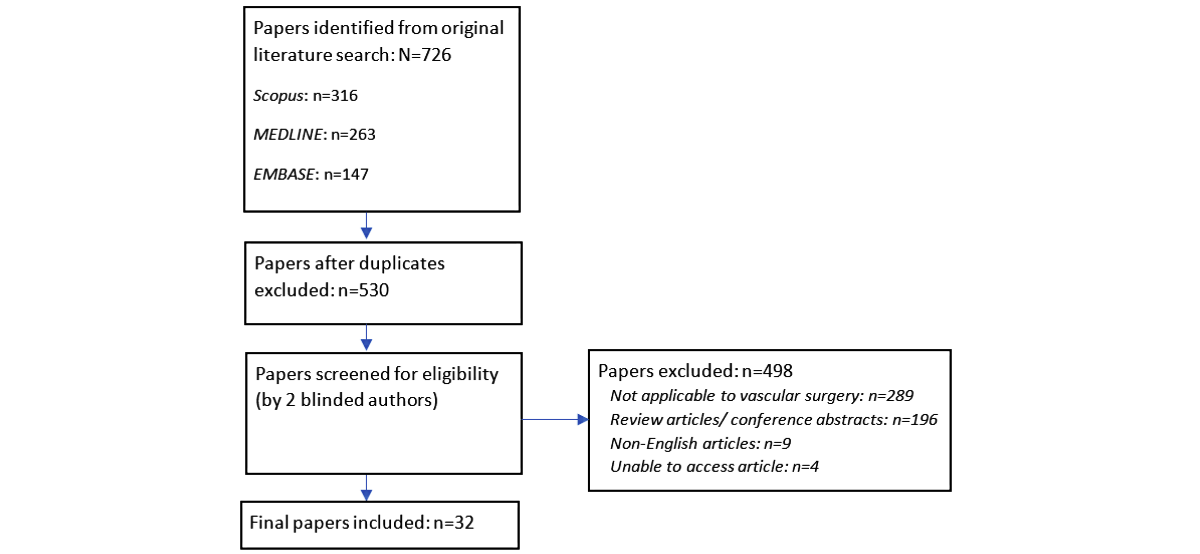
Search strategy. Search of MEDLINE, Scopus, and Embase databases. Exclusions shown.

A total of 726 articles were identified from the initial search across the 3 databases, with 32 (4.4%) meeting the inclusion and exclusion criteria and being included in the final results. Please see Figure 1 for detailed information, including the reasons for article exclusion.

### Characteristics of the Sources of Evidence

A total of 32 articles applicable to vascular and endovascular surgery met the criteria for inclusion. The included articles were sorted into three categories: open vascular surgery, endovascular surgery (subdivided into aortic, visceral, peripheral, and venous, where applicable), and training (Multimedia Appendix 1 [6,15-45]). Many studies were preclinical in application, with 44% (14/32) including human study populations with limited numbers. There were no human studies with equivalent outcome measures for comparative quantitative data analysis.

### Critical Appraisal

Quality assessment could only be performed in cases where human study populations were evaluated, which was in 44% (14/32) of the studies. The Cochrane risk of bias assessment was not used as no randomized trials were included in our review. Among the clinical studies, 6% (2/32) were cohort studies [15,19], which were found to have a moderate risk of bias according to the Cochrane risk of bias tool for nonrandomized trials [13]. Of the 32 studies, 1 (3%) was a case-control study [25] with a score of 55% according to the Joanna Briggs Institute Critical Appraisal Checklist, 7 (22%) were case series studies [28,32,37-40] with SD (16.1%) an average score of 70% (SD 16.1%; range 50%-80%), and 3 (9%) were case reports [16,36,39] an average score of 65% (range 40%-95%).

The articles were further subdivided into studies that focused on *concept and design* to reflect the early stage of this technology in vascular surgery and those that conducted research directly *applied* to surgery, training, or simulation.

### Synthesis of Results

#### Overview

The proportion of studies related to concept development of AR was 38% (12/32), whereas the rest were applied to clinical and simulation contexts. Of the 32 studies, 29 (91%) involved deriving digital structures of blood vessels from clinical imaging (Multimedia Appendix 2 [6,15-45]), including noncontrast computed tomography (CT), CT angiography, cone-beam CT (CBCT), magnetic resonance angiography, conventional ultrasound (US), intravascular US, and 3D US.

Virtual data are processed into a virtual object and displayed to the clinician on a screen, projector, monitor, or AR HMD. Several steps are required to complete processing, including segmentation to reduce the model to the area of interest (eg, aorta), often by thresholding via a Hounsfield scale, for example, and then using a 3D computer-generated surface mesh, refining the mesh by filling holes or defects into a model and sometimes using a slicer to break up the model into its different anatomical sections [41].

#### Registration and Tracking

Registration and tracking are essential components of AR, allowing for the alignment of virtual and real data in a usable way. Registration is commonly performed using markers, where a particular pattern or anatomical landmark in the real world is used as a reference corresponding to a virtual marker derived from medical imaging [10]. This can be performed manually or using trackers. Marker-less tracking is now possible owing to technological advances that correlate patterns in real and virtual data in real time [46,47]. Integrated or external optical sensors (infrared or color) can be used to track markers or recognize patterns from a patient’s anatomy. In recent years, Red, Green, Blue-Depth cameras that track both depth and color simultaneously have allowed for the contemporaneous tracking of the real world [48]. Electromagnetic tracking is a useful method for tracking instruments deep below the surface [26]. In many cases, such as with Microsoft HoloLens, a variety of sensor inputs are used for hybrid tracking [49].

Challenges with these techniques include the deformability of the anatomy; for example, when stiff endovascular devices are inserted into blood vessels. These changes can cause registration errors, which can be clinically significant, for example, with complex visceral anatomy in endovascular aneurysm repair (EVAR). Groher et al [24] proposed computational algorithms to allow for some accurate deformability of 3D models for registration with 2D fluoroscopy. Another innovative tracking solution combating this problem involves electromagnetic trackers on the tips of angiography catheters, which can provide accurate information on the 3D position and orientation of the catheter in space, such as those used by Garcia-Vasquez et al [27].

Alternatively, intravascular US on the tip of catheters can be used to create a 3D model of blood vessels during endovascular procedures with magnetic trackers to orientate the catheter in real time, as proposed by Shi et al [29]. Parrini et al [21] tested the use of freehand external magnets as a means of guiding magnetic endovascular devices to their targets using AR vessel models on a phantom.

#### Endovascular Procedures

Given the inherent reliance of endovascular procedures on imaging, the potential benefits of AR were identified early, in particular to improve clinicians’ understanding of 3D anatomy during critical steps as well as to reduce time and radiation and contrast exposure [50]. Image overlay techniques are used for complex endovascular work worldwide and usually rely on merging live fluoroscopy with 2D or 3D images from x-ray, CT, or magnetic resonance imaging.

#### EVAR and Aortic Disease

EVAR is increasingly being performed for the repair of both complex and conventional infrarenal abdominal aortic aneurysms in the elective and emergency settings.

AR visualization has now been used in planning and EVAR navigation, with a case report by Rynio et al [16] describing the use of an AR HMD to project an aortic aneurysm and bones (vertebral column lying posterior to the aneurysm) as a 3D hologram that responds to gestures and voice commands.

A “3D road map” published in 2013 was developed by Fukuda et al [34] using preoperative CT imaging, where bone marrow subtraction and the use of the iliac crest and lumbar vertebrae as landmarks resulted in the creation of an image overlay to guide aortic endografting. This reduced the need for digital subtraction angiography (DSA) and its associated risks, ultimately giving rise to fusion imaging.

Fusion imaging has been used for endovascular navigation in hybrid vascular operating theaters, particularly in complex fenestrated EVARs. This uses preoperative 3D CT imaging to overlay vascular structures onto perioperative 2D fluoroscopy images with the aim of reducing fluoroscopy time, contrast agent dosing, and overall operating time. This can also be used in a conventional operating theater with a mobile C-arm with good results for conventional aortic endografting, as reported by Kaladji et al [25]. Koutouzi et al [32] modified this modality to require carbon dioxide DSA to confirm accurate registration of the CT-derived 3D vascular overlay to avoid iodinated contrast in patients with poor renal function or contrast allergies.

**Figure 2:**
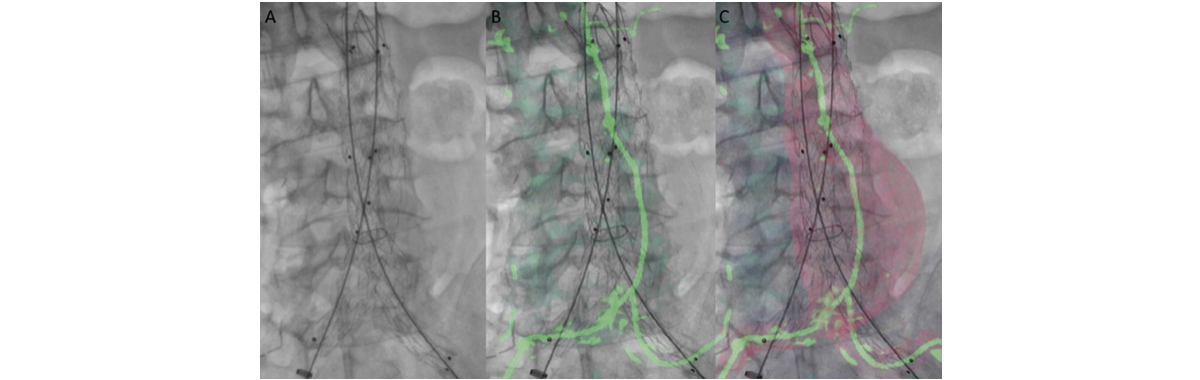
During the procedure, different information was overlaid onto the native 2D fluoroscopic image to guide instrument placement: (A) none, (B) centerlines and key points can be projected, and (C) artificially enhanced aortic volume. Reprinted from European Journal of Vascular & Endovascular Surgery, 49/3, Kaladji A, Dumenil A, Mahé G, Castro M, Cardon A, Lucas A, Haigron P, Safety and accuracy of endovascular aneurysm repair without pre-operative and intra-operative contrast agent, 255-261, 2015, with permission from Elsevier [33].

Kaladji et al [33] went on to successfully demonstrate the use of fusion imaging to perform EVAR without the use of any iodinated contrast in the pre- or perioperative phase in a case series of 6 patients. Patients with severe chronic kidney disease underwent unenhanced CT imaging. Centerlines for EVAR planning were manually drawn, and key anatomical points or landing zones were marked following segmentation and processing. A preoperative 3D image overlay reconstruction with these markers was then projected onto 2D fluoroscopy imaging to guide the placement of the aortic endograft without the use of a contrast agent (Figure 2). No endoleaks were noted during postoperative duplex surveillance, with minimal error in the positioning of the aortic endograft on postoperative CT imaging and no significant reduction in renal function.

A series of 101 patients by Schulz et al [19] in 2016 tested the use of image overlay alone in patients undergoing conventional infrarenal EVAR. Fusion image overlay using images from preoperative CT angiograms with images obtained from an intraoperative CBCT was compared with intraoperative DSA. Although <5-mm accuracy was observed in most patients (68% of the patient cohort), significant deviations were noted in some patients, including significant caudal deviation in 9 patients (9% of the patient cohort), which would have resulted in coverage of the lowest-lying renal artery, resulting in significant operative morbidity. Therefore, a contrast injection study for cannulation of the lowest-lying visceral vessel was recommended.

Koutouzi et al [28] described a technique that aims to use image overlay to reduce the risk of covering intercostal arteries during thoracic EVAR (TEVAR), which may affect spinal cord perfusion, causing paraparesis. Intraoperative CBCT was used to perform 3D-3D registration with a preoperative CT angiogram to enhance accuracy. Once calibrated onto live fluoroscopy, this overlaid 3D model was used to guide the TEVAR and avoid preplanned intercostal arteries. A case series of 7 was reported without spinal cord injury.

**Figure 3:**
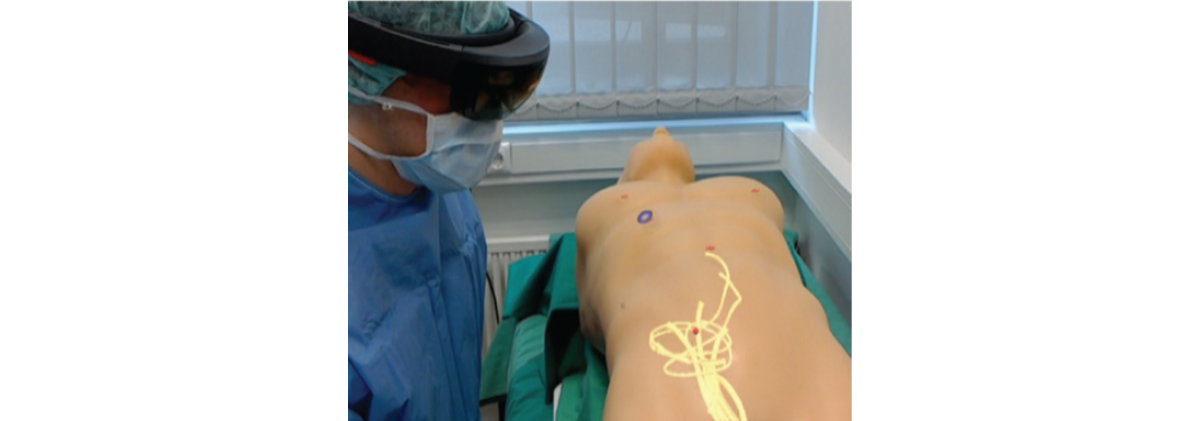
View from HoloLens display of 3D vasculature from 3D ultrasound being projected onto a phantom. Reproduced from García-Vázquez V et al [27] under the Creative Commons Attribution Non-Commercial License.

A common problem with image overlay techniques is the nondeformability of a rigid 3D model created from a preoperative CT scan. Garcia-Vasquez et al [27] attempted to overcome this problem using 3D US to create a real-time model of the patients’ anatomy that was visible to a clinician wearing the AR HMD. Coupled with the electromagnetic catheter tip tracking system used by von Haxthausen et al [26], this concept has been proposed to perform EVAR completely free of radiation and intravenous contrast and tested on a phantom model with a 3D model of an aortic aneurysm [27]. The concept showed promising progress (Figure 3); however, issues around registration accuracy remain.

#### Endovascular—Peripheral Arterial Disease

Endovascular techniques are being applied to increasingly complex surgical problems in patients with comorbidities. The potential to reduce radiation and contrast exposure while navigating tortuous vessels makes the application of AR technology more attractive.

Lu et al [36] described successful retrograde peroneal access through an AR system with CT angiogram image–derived overlay of fluoroscopic images. AR glasses were worn by the operator to guide the needle trajectory. The case report highlighted technical difficulties, including the need for manual registration and having to align virtual dots on their AR HMD with fiducial markers on the patient’s leg. In addition, aligning the trajectory of the needle path on the AR HMD required the acquisition of new technical skills.

The work of Goudeketting et al [37] focused on the accuracy of image fusion based on preprocedural contrast-enhanced magnetic resonance angiography during percutaneous angioplasty of iliac lesions. They found that guidewires and endovascular catheters did not cause significant vessel displacement to influence image fusion according to angiographic experts.

Swerdlow et al [15] retrospectively compared carotid stenting with 2D-3D image fusion (46 patients) and without (70 patients). Magnetic resonance imaging or CT angiography images were overlaid onto the real-time 2D fluoroscopy. They observed significantly improved cannulation times and reduced radiation exposure using this technique.

#### Endovascular—Venous Disease

Endovascular venous intervention, compared with arterial intervention, can be more challenging because of the lack of landmarks from vessel calcification and reduced vessel wall thickness. Schwein et al [38] used image fusion techniques with magnetic resonance venography to successfully recanalize 4 patients with central venous occlusion. It was felt that magnetic resonance venography image fusion improved clinician confidence and the safety of difficult venous endovascular navigation.

### Open Surgery

#### Overview

“Traditional” open vascular surgery has increasingly been replaced by novel endovascular techniques, which, in combination with problems with tissue deformation, as previously described, may explain why AR is less well researched in this field.

**Figure 4:**
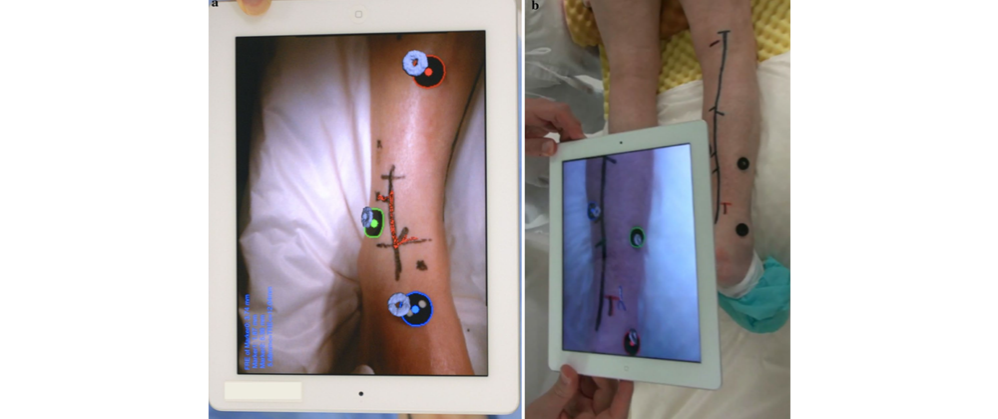
Computed tomography–derived 3D model is superimposed onto camera images. Doughnut-shaped fiducial markers on the limb help match images so that the target artery and its branches can be overlaid onto the limb as red (A) or blue (B) lines. Reprinted by permission from the Springer Nature Customer Service Centre GmbH: Springer Nature. New simple image overlay system using a tablet PC for pinpoint identification of the appropriate site for anastomosis in peripheral arterial reconstruction. Mochizuki Y, Hosaka A, Kamiuchi H, Nie JX, Masamune K, Hoshina K, et al. 2016 [40].

The use of a new image overlay system has been demonstrated successfully in lower limb revascularization surgery by Mochazuki et al [40]. This system used preoperative CT imaging to help locate the target distal anastomotic site and plan a limited incision accordingly using a branch artery as a reference point (Figure 4).

**Figure 5:**
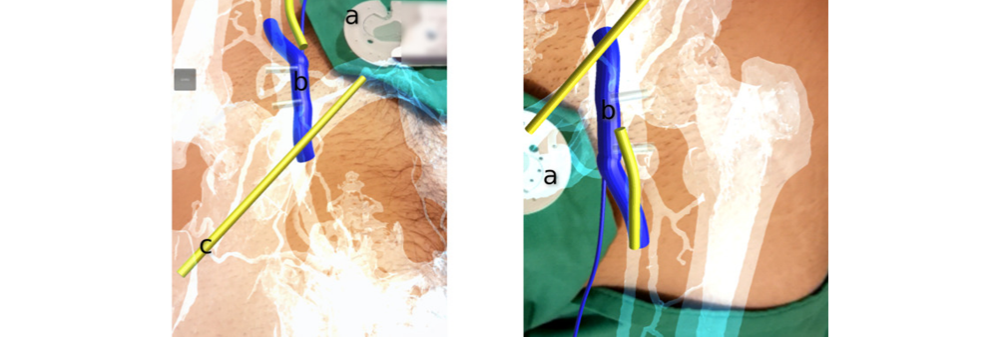
Screen capture of augmented reality–assisted surgery system developed by Aly [6]. Fiducial marker (sterile suture pack) used for registration and tracking with 3D model derived from computed tomography angiogram superimposed from mobile phone. (A) Tracking marker, (B) common femoral vein, and (C) inguinal ligament. Reproduced under the Creative Commons Attribution License CC-BY 4.0.

Modern-day mobile phones have multiple sensors that may be used for AR-assisted surgery. The benefits of easy accessibility (most operators have access to a mobile phone) and cost make them an attractive option. An AR-assisted surgery system was developed by Aly [6] to provide 3D guidance during vascular procedures. CT angiogram–derived images were used by a smartphone to produce a 3D guidance model fused with the patient’s anatomy, and rotational and positional tracking were compared for planning an iliofemoral bypass as well as for an endovascular sheath placement in the common femoral artery (Figure 5).

#### Robotic Surgery

The use of AR with robot-assisted surgery is of increasing academic interest worldwide. Pietrabissa et al [39] successfully used an AR HMD to superimpose a surgical anatomy from CT angiography onto the patient’s body for preoperative planning to guide trocar placement and dissection so as to minimize disruption using the da Vinci surgical system. Although robot-assisted surgery is becoming increasingly common for vascular interventions, the use of AR is in the early stages of adoption and investigation.

#### Training and Simulation

Published studies on AR vascular and endovascular training were limited in our review to 16% (5/32) of the papers. Of these 5 papers, 1 (20%) applied this to training vascular surgeons, whereas 4 (80%) focused on design concepts.

Mangina et al [41] tested the concept of creating a 3D model of the aorta from CT angiography images using a number of programming tools and platforms, including *Unity*, *ARToolKit*, and *Pro/ENGINEER*. A model aorta was created with the potential for merging with VR and AR tracking technologies to create an accurate training and educational tool for application to both open and endovascular training.

Bartesaghi et al [43] used similar techniques to create a 3D model of the aorta projected onto a mannequin to allow for 3D AR simulation of an EVAR. The original model was derived from CT angiography images, and segmentation with meshing was performed to create the model. The software was optimized for the interplay between the simulation and a hardware mannequin, allowing surgical trainees to learn the steps of an EVAR.

**Figure 6:**
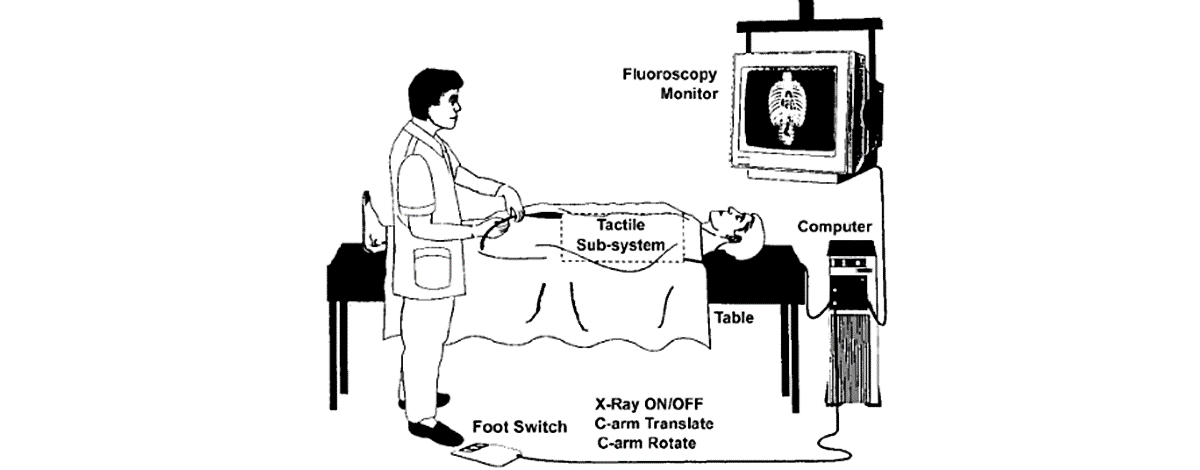
Early augmented reality interventional simulator design. Reproduced from Anderson et al [45], with permission from © Georg Thieme Verlag KG. Note: The permission of the figure stays with the publisher, and any further reuse will need explicit permission from the publisher.

Similarly, Anderson et al [45] described the development of a PC-based AR simulation system for interventional radiology (Figure 6). This system allows for real-time manipulation of catheters and guidewires through a tactile user interface device that creates hand-eye coordinated realism of the movement of instruments over 3D-generated models of vessels. Extra enhancements have been built in, including the administration and subsequent washout of contrast and control of the C-arm. The paper reports good initial feedback from neurological and peripheral vascular interventionalists who felt it had 75% of the desired functionality and was useful for educational simulation, with further work required to achieve sufficient realism for preoperative planning.

Crowley et al [42,51] built on the work of Mangina et al [41], further exploring the concept of EVAR MR simulation. They found that the creation of a solid 3D-printed model of an aortic aneurysm through which real instruments (guidewires, catheters, and delivery devices) can be placed and manipulated gave the simulation tactile realism. A mobile device provides the user with fluoroscopic data through an AR software (ARToolKit or Daqri) while haptic feedback is provided by the 3D model. Limitations of 3D-printed models include the lack of vascular wall deformability and the realism of feedback from catheter tips against the model wall. The system’s usability was tested by medical students and university lecturers.

An AR system for US-guided endovascular surgical training was devised by Cheng et al [20]. Live US images were transferred to a prescanned phantom model of the aorta and iliac arteries, which was extended to a computer-assisted remote endovascular surgery model. The user is able to navigate an endovascular catheter using a robotic device with computer-generated images to augment the 3D understanding of the catheter in space. The phantom provides both haptic and visual feedback aiming to be as realistic as possible.

Rudarakanchana et al [44] applied the concept of simulation to the pressured setting of ruptured EVAR. This was achieved through VR simulators being integrated into a simulated angio suite with the whole team present. A total of 10 teams were tested: 5 led by interventional specialists and 5 led by trainees. This study measured the time to achieve proximal control and total procedure and fluoroscopy times. Experts were significantly faster than trainees using reduced fluoroscopy time, suggesting that the simulation model had good applicability to real-life experience. In particular, the value of the simulation for improving team communication and human factor skills was highlighted through feedback, suggesting that AR and MR simulation could be useful adjuncts to improving ruptured EVAR outcomes.

Our review demonstrates that the most used modality for obtaining data for display is CT angiography (Table 1), with CBCT often used in the registration process with the patient in the operating suite. The type of display will depend on the application, and a range of display types were included, with image fusion studies making monitors the most prevalent display (Table 2).

**Table 1:** Most of the included studies derived data from 3D clinical imaging, which can be overlaid onto the real world (N=32).

Data source	Studies, n (%)
Noncontrast CT^a^	1 (3)
CT angiography	22 (69)
Cone-beam CT	11 (34)
MR^b^ angiography or venography	3 (9)
Conventional US^c^	1 (3)
Intravascular US	1 (3)
3D US	2 (6)
Rotational XR^d^	1 (3)
Computer-generated model	3 (9)

^a^CT: computed tomography.

^b^MR: magnetic resonance.

^c^US: ultrasound.

^d^XR: x-ray.

**Table 2:** Types of augmented reality (AR) displays. A monitor was the most frequently used type of display, often used for image fusion in interventional procedures (N=32).

Types of AR display	Studies, n (%)
AR HMD^a^ (including HoloLens)	9 (28)
Monitor	20 (63)
Mobile phone	2 (6)
Projector	1 (3)

^a^HMD: head-mounted display.

## Discussion

### Summary of Evidence

The reviewed studies demonstrate that AR in surgery often relies on the registration of a virtual image or object onto the real patient using a tracking method, which can trace the real environment and place virtual objects in the correct position and orientation. Our study revealed a wide range of applications of AR in vascular surgery. Image overlay technology is increasingly used, with its value being reported in endovascular treatment of peripheral arterial disease [37], deep venous disease [38], carotid stenting [15], and aortic disease (EVAR [19,32], TEVAR [28], and complex EVAR cases [31,52]). There is evidence that it can improve technical success while reducing procedural times, radiation dose, and contrast volume.

However, concerns remain regarding the accuracy of image registration, especially with more complex anatomies [19]. A common problem is the deformation of vascular structures with respiration, surgical manipulation, or *stretching out* of vessels (such as iliac and target visceral arteries for EVAR) with rigid stent delivery systems. Emerging technologies offer novel solutions for improving the real-time accuracy of overlaid data. Robust deformable registration algorithms or intraoperative real-time 3D scanners are potential solutions to assist AR-guided surgery on soft or deformable structures. In particular, 3D and intravascular US offer radiation- and contrast-free modalities to create real-time images for intraoperative use [27], whereas electromagnetic tracking provides contemporaneous spatial information about catheter devices [26]. The integration of these technologies demands high levels of data transmission and processing, which must be met by technology to prevent lag [53].

AR HMDs allow the wearer hands-free access to virtual data overlaid directly onto the patient. Several studies with EVAR in particular have shown early promise that they can reduce the need for radiation and contrast exposure while improving accuracy [26,27,31]. Studies on open and robotic surgery corroborate this [39,54]. AR HMDs such as HoloLens still have a few technical concerns that could restrict their usefulness. Their weight (500-645 g depending on the device and manufacturer) can cause discomfort and fatigue during long procedures. AR HMD processing capacity and memory are limited, which can restrict certain applications. The immersive experience can be limited by a restricted field of view and projection size, whereas image quality and computational time will improve as the technology evolves. There is no consensus yet on the optimal methods for merging and displaying virtual and real information so that depth perception or focus and visual clutter do not distract the wearer [11,55].

Most of the included studies (28/32, 88%) described small-scale implementations of AR with limited study participants and a wide range of AR hardware and software platforms in their research. Only 44% (14/32) of the studies included human study populations. As AR becomes more ubiquitous for vascular surgery, more evidence will become available, and we believe that these limitations will pose less of an issue.

A significant proportion of the studies were preclinical in their applications and demonstrated proof-of-concept findings. Nevertheless, AR solutions are applicable to a wide spectrum of fields in vascular surgery, including those highlighted in this scoping review.

### Limitations

Three databases (MEDLINE, Embase, and Scopus) were searched for this review, allowing for a broad assessment of the current impact of AR clinically and educationally while also scoping potential future applications. However, in this fast-moving field, AR applications under development by private enterprises were not included in the contents of our review. Although our selection criteria focused on vascular and endovascular surgery, there is significant crossover with work applied to other medical and surgical specialties, and wider development within interventional radiology and allied surgical specialties will further inform applications within vascular and endovascular surgery.

### Conclusions

AR has shown potential to enhance accuracy and reduce procedural time, radiation exposure, and contrast dose in a range of vascular surgery applications by digitally augmenting the clinicians’ procedural ability, reducing surgical risk, and improving patient outcomes. Clinicians, who demand high levels of accuracy and patient safety, should be aware of potential technical pitfalls when using AR technology on patients but should also be aware of the potential benefits. AR has also been shown to be an increasingly valuable tool in surgical simulation and education. As technology improves, it can be expected that AR will become increasingly relied upon as an arrow in the surgical quiver and that more applications will be found for AR, which could benefit clinicians. Future development and studies should assess whether the use of AR affords improvements in patient experience and in clinical effectiveness as objective measures of improvement in outcomes and cost-effectiveness of this technology.

## Appendix

Multimedia Appendix 1 Studies applicable to augmented reality in vascular and endovascular surgery.

Multimedia Appendix 2 Imaging, registration, and display types within review.

## Abbreviations

AR: augmented reality
CBCT: cone-beam computed tomography
CT: computed tomography
DSA: digital subtraction angiography
EVAR: endovascular aneurysm repair
HMD: head-mounted display
MR: mixed reality
PRISMA: Preferred Reporting Items for Systematic Reviews and Meta-Analyses
PRISMA-ScR: Preferred Reporting Items for Systematic Reviews and Meta-Analyses extension for Scoping Reviews
PROSPERO: international prospective register of systematic reviews
TEVAR: thoracic endovascular aneurysm repair
US: ultrasound
VR: virtual reality
