# Supplementary material for: Augmented Reality in Vascular and Endovascular Surgery: Scoping Review
Source: JMIR Serious Games. 2022 Sep 23;10(3):e34501. doi: 10.2196/34501 (PMC9547335; doi:10.2196/34501)
Supplement: Multimedia Appendix 1 [file games_v10i3e34501_app1.docx]

**Multimedia Appendix 1.** Studies applicable to augmented reality in vascular and endovascular surgery.

| ***Article author*** | ***Title*** | ***Category*** | ***Clinical study*** *(No. patients)* | ***Risk of Bias*** *(Human studies)* | ***Patient/ participant sample*** | ***Method and Outcome*** |
| --- | --- | --- | --- | --- | --- | --- |
| **Swerdlow N.J *et al*. 2019** | Three-dimensional image fusion is associated with lower radiation exposure and shorter time to carotid cannulation during carotid artery stenting | Endovascular | Cohort (116) | Moderate  (ROBIN-1) | 116 transfemoral CAS patients between 2009 and 2017. No difference in characteristics between control and image fusion cohorts | *CTA images were used to create a 3D roadmap to assist carotid cannulation. This was registered with CBCT intraoperatively. Outcome* Carotid artery stenting with 3D image fusion was associated with lower radiation exposure, shorter time to CCA cannulation and shorter procedures. |
| **Rynio P *et al*. 2019** | Holographically-Guided Endovascular Aneurysm Repair | Endovascular | Case report (1) | 40% (JBI CAC*) | 77M with Abdominal aortic aneurysm | *Aneurysm and bones (derived from CTA) projected onto fluoroscopic image using HMD in case of 77M undergoing EVAR. Manual registration. Outcome* Hologram useful during EVAR. Limitations with binocular vision, battery life, inability to manipulate hologram |
| **Grinshpoon A *et al.* 2018** | Hands-free augmented reality for vascular interventions | Endovascular | N/A | N/A | N/A | 3D user-interface using HoloLens to allow head-movements and voice commands to control model *Outcome* Successful design of user interface for use with Hololens. |
| **Kilian-Meneghin J *et al*. 2018** | Evaluation of Methods of Displaying the Real-Time Scattered Radiation Distribution during Fluoroscopically-Guided Interventions for Staff Dose Reduction | Endovascular | N/A | N/A | N/A | Microsoft Kinect camera and fusion system used to display ionising radiation dose with augmented reality. *Outcome* Questionnaire confirmation of usefulness to staff as a guide for positioning and a reminder of ionising radiation |
| **Schulz CJ *et al*. 2016** | Fusion Imaging to Support Endovascular Aneurysm Repair Using 3D-3D Registration | Endovascular | Cohort (101) | Moderate  (ROBIN-1) | EVAR patients, median age 72, 93 men | 3D-3D fusion using intraoperative nCBCT overlaid with CTA images. Accuracy measured as difference between lowest renal artery and fusion imagine. *Outcome* Craniocaudal accuracy was achieved in 68% of cases, however due to risk of deviations, noncontrast EVAR without confirmation of FI overlay with contrast was not recommended. |
| **Cheng I *et al.* 2014** | An augmented reality framework for optimization of computer assisted navigation in endovascular surgery | Endovascular | N/A | N/A | N/A | Concept prototype of augmented reality phantom for training, using US images, GPS and EM tracking *Outcome* Successful creation of integrated simulation of endovascular procedures |
| **Parrini S *et al.* 2014** | Augmented reality system for freehand guide of magnetic endovascular devices | Endovascular | N/A | N/A | 10 operators (unclear background) for concept prototype | HMD used to guide endovascular capsules using extracorporeal magnets in a phantom study. *Outcome* Success rates on low fidelity phantom across three different paths were 90% with good feedback. |
| **Van Den Berg J.C. 2013** | Three-dimensional image overlay to assist endovascular procedures | Endovascular | N/A | N/A | N/A | Technical considerations for use of 3D image fusion and likely application for complex procedures |
| **Wang J *et al.* 2012** | Augmented reality during angiography: Integration of a virtual mirror for improved 2D/3D visualization | Endovascular | N/A | N/A | 24 participants. 10 clinicians, 5 R&D engineers, 9 computer scientists. | Overlay of mirror image onto fluoroscopic images to improve 3D visualisation. *Outcome* Positive feedback from participants |
| **Groher M *et al*. 2009** | Deformable 2D-3D registration of vascular structures in a one view scenario | Endovascular | N/A | N/A | N/A | Concept and complex algorithm capable of registering deformable vessels in a 3D projection. *Outcome* The performance of the proposed method was tested quantitatively on synthetic examples as well as real angiographic scenes, with improved positioning relative to rigid non-deformable |
| **Kaladji *et al*. 2019** | Fusion Imaging for EVAR with Mobile C-arm | Endovascular – Aortic | Case-control (54) | 55% (JBI CAC*) | Prospectively recruited 54 patients (49 men, 92.6%) of mean age 73.4. Mean BMI was 28.3 | Prospective case-control study using EndoNaut image fusion system, with mobile C-arm, of pre-operative CTA onto live fluoroscopic images.  *Outcome* Similar operation times with reduced contrast agent <0.0001. Feasible to use fusion imaging with mobile c-arm. |
| **Haxthausen F.V *et al*. 2019** | Catheter pose-dependent virtual angioscopy images visualized on augmented reality glasses | Endovascular- Aortic | Feasibility study | N/A | N/A | EM tracking of angiography catheter with Microsoft HoloLens to create virtual angioscopy images in phantom model. *Outcome* Feasibility of EM tracking of catheter tip with 3D model demonstrated on phantom with mean latency time of 11 milliseconds. Rigid phantom does not mimic real-life vascular deformability |
| **García-Vázquez *et al*. 2018** | Navigation and visualisation with HoloLens in endovascular aortic repair | Endovascular – Aortic | Feasibility study | N/A | N/A | Phantom prototype design using 3D US and electromagnetic tracking devices (catheter-tip sensor) with Microsoft HoloLens to guide EVAR procedure. .  *Outcome* Promising conceptstudy on US model to overcoming need for radiation/ contrast during EVAR. Drawbacks include latency time (259 seconds) non-deformability, and limited information with sensor at catheter tip. Catheter with 7 sensors provides more realistic information. |
| **Koutouzi *et al*. 2017** | 3D Image Fusion to Localise Intercostal Arteries During TEVAR | Endovascular- Aortic | Case series (7) | 65% (JBI CAC*) | 6 men, mean age 66.7. All requiring TEVAR | 3D-3D image fusion of preoperative CTA and intraoperative nCBCT overlayed with fluoroscopy. *Outcome* Successfully used in 7 procedures. Able to spare pre-operatively determined intercostal arteries. Overlay not always accurate and should be checked with angiography in high precision cases |
| **Chaoyang Shi *et al. 2016*** | In vitro three-dimensional aortic vasculature modelling based on sensor fusion between intravascular ultrasound and magnetic tracker | Endovascular- Aortic | Feasibility study | N/A | N/A | Proof of concept prototype using EM tracking with intravascular ultrasound to create live 3D aortic vascular modelling for EVAR. *Outcome* Prototype built and tested with promising results. Challenges for implementationin real-time due to processing lag. |
| **Rolls A.E *et al*. 2016** | A Comparison of Accuracy of Image- versus Hardware-based Tracking Technologies in 3D Fusion in Aortic Endografting | Endovascular- Aortic | Observational (12) | 95% (JBI CAC*) | 12 patients with AAA of varying morphology. 11 male, 4 DM, mean BMI 26.7 | Prospective trial involving 12 patients who received with preoperative CTA and intra operative CBCT fusion overlaid with fluoroscopic images using both HBT and IBT technologies for EVAR. *Outcome* Both modalities compared for error. Significant reduction in overlay error using image-based tracking. |
| **Dumenil A *et al*. 2016** | A versatile intensity-based 3D/2D rigid registration compatible with mobile C-arm for endovascular treatment of abdominal aortic aneurysm | Endovascular – Aortic | Feasibility study | N/A | Retrospective registration of pre-operative CTA and peri-operative fluoroscopy for 26 patients | Concept testing of mage fusion technique with reduced registration timecompatible with a mobile C-arm. *Outcome* registration system compatible with EVAR workflow and can be used throughout procedure |
| **Koutouzi *et al*. 2015** | EVAR Guided by 3D Image Fusion and CO2 DSA: A New Imaging Combination for Patients With Renal Insufficiency | Endovascular – Aortic | Case Series (3) | 50% (JBI CAC*) | 75, 76 and 81 year old men with CKD. One juxtarenal, one infrarenal AAA, one EIA aneurysm | Pre-operative CTA, intraoperative nCBCT fused, and registration confirmed intraoperatively with C02 DSA. *Outcome* Reduces iodine-contrast exposure. Contrast fluoroscopy advised to verify placement intraoperatively due to small registration errors. |
| **Dumenil A *et al*. 2015** | Safety and accuracy of endovascular aneurysm repair without pre-operative and intra-operative contrast agent | Endovascular- Aortic | Case Series (6) | 80% (JBI CAC*) | 6 patients (mean age 77.1) underwent EVAR (5 AAA and 1 thoracic). Median eGFR 17. | Preoperative non-enhanced CT optimised/ extracted and overlaid onto live 2D fluoroscopic images with intraoperative nCBCT for patients with renal failure. *Outcome* Successful aneurysm repair on 6 patients without use of contrast, with good placement confirmed on post-operative CT and intraoperative duplex |
| **Fukuda T *et al*. 2013** | Evaluation of automated 2D-3D image overlay system utilizing subtraction of bone marrow image for EVAR: Feasibility study | Endovascular- Aortic | Case series (20) | 75 % (JBI CAC*) | 20 patients undergoing EVAR, 18 male, mean age 79.7, BMI 22.8. | Enhanced preoperative CTA and intraoperative CBCT fused with fluoroscopic onlay. Contrast injection to check accuracy and as final check. *Outcome*  Series of 20 patients confirm feasibility of ‘3D roadmap’ use in EVAR to reduce maneuvers, contrast and radiation required. 80% accuracy of registration of iliac bifurcation and renal arteries despite deformation with insertion of instruments. |
| **Carrell *et al*. 2010** | Feasibility and limitations of an automated 2D-3D rigid image registration system for complex endovascular aortic procedures | Endovascular – Aortic | Accuracy Study | N/A | Retrospective analysis of 11 patients undergoing aortic repair. | An automated 2D-3D image registration system was used to overlay preoperative CTA and fluoroscopic images retrospectively. *Outcome* Strong correlation between registration errors (mean 6.5mm) in highly angulated aortas (= or <30 degrees) due to deformation with device insertion Mean error 2.5mm in less angulated aortas.(<30 degrees) p<0.0001) |
| **Lu W *et al*. 2020** | Augmented reality navigation to assist retrograde peroneal access for the endovascular treatment of critical limb ischemia | Endovascular- Peripheral | Case report (1) | 88% (JBI CAC*) | 84F right CLI | HMD used to facilitate retrograde peroneal access of tibioperoneal trunk with CTA assisted augmented reality.  *Outcome* Successful and feasible approach. Learning curve for user to ensure overlapping of markers and to follow AR projected trajectory |
| **Goudeketting *et al*. 2018** | The use of 3D image fusion for percutaneous transluminal angioplasty and stenting of iliac artery obstructions: validation of the technique and systematic review of literature | Endovascular – Peripheral | Case series (11) | 65% (JBI CAC*) | 11 patients undergoing iliac PTA | 3D Image fusion with pre-operative CE-MRA and nCBCT with fluoroscopic images performed and accuracy measured.  *Outcome* Accurate fusion, with minimal displacement (mean 4..5 +/- 2.5mm) of iliac arteries caused by insertion of catheters (minimal difference in displacement between catheterized and non-catheterised iliac arteries) |
| **Schwein *et al*. 2017** | Magnetic resonance venography and three-dimensional image fusion guidance provide a novel paradigm for endovascular recanalization of chronic central venous occlusion | Endovascular – Venous | Case Series (4) | 60% (JBI CAC*) | 4 patients with previously failed standard fluoroscopic endovascular cannulation for central venous occlusion | MRV and intra-operative nCBCT image fusion with fluoroscopic overlay. *Outcome* Successfully crossed occlusion in all patients without complication. Image fusion feasible for stenting central venous occlusion and could improve success, safety and surgical confidence |
| **Pietrabissa A *et al*. 2009** | Mixed reality for robotic treatment of a splenic artery aneurysm | Endovascular – Visceral | Case report (1) | 80% (JBI CAC*) | Patient with 3cm splenic aneurysm | HMD with pre-operative CTA derived 3D model allowing targeted trocar placement and localized robotic dissection  *Outcome* Successful robotic exclusion of a splenic artery aneurysm assisted by AR with good surgeon feedback. |
| **Aly, Omar 2020** | Assisting Vascular Surgery with Smartphone Augmented Reality | Open | Feasibility Study | N/A | N/A | An augmented reality assisted surgery (ARAS) system was developed using a smartphone and CTA derived 3D models. Two types of optical tracking were tested for stability. *Outcome* Good initial results from combined optical tracking with gyroscopic data. Limitations of lack of deformability of model. Good feedback and improved reported usability and reduced discomfort compared to HMD |
| **Mochizuki *et al. 2016*** | New simple image overlay system using a tablet PC for pinpoint identification of the appropriate site for anastomosis in peripheral arterial reconstruction | Open | Case series (11) | 75% (JBI CAC*) | 10 patients, 11 limbs. Mean age 69. | CTA enhanced to identify distal target vessel. US used to confirm intraoperatively and image fusion with intraoperative augmented reality to identify vessel, using tablet display *Outcome* Successful identification of 11 target branches. One missed by 10mm, due to incorrect marker tracking |
| **Mangina E *et al*. 2018** | 3D modelling for augmented reality systems in novel vascular models | Training and simulation | Concept design | N/A | N/A | Using opensource software inc. UNITY and PROENGINEER, a 3D model was created and sliced for use in VR or AR applications.  *Outcome* Successful creation of 3D model of AAA from CTA using open source software |
| **Burke E *et al*. 2017** | Augmented reality EVAR training in mixed reality educational space | Training and simulation | N/A | N/A | N/A | AR EVAR simulation prototype using CTA derived- 3D printed vessels registered with a ‘fluoroscopic display’ on a mobile phone.  *Outcome* Good initial user feedback. Further development required for realism. HMD devices to be used to improve user experience |
| **Bartesaghi S *et al*. 2017** | Spatial augmented reality and simulations to improve abdominal aortic aneurysm diagnosis and monitoring | Training and simulation | Feasibility Study | N/A | N/A | Proposed techniques for computer aided design models of AAA from CTA data, and evaluation of potential uses when combined with computational flow dynamics. Proposed model for simulation of EVAR.*Outcome* Mixed reality simulation, with successfully created 3D model from CTA which was projected onto a mannequin for live simulated EVAR. |
| **Rudarakanchana N *et al*. 2014** | Endovascular repair of ruptured abdominal aortic aneurysm: Technical and team training in an immersive virtual reality environment | Training and simulation | Feasibility study | N/A | 5 consultant and 5 trainee endovascular specialists | Virtual reality simulators were integrated into a simulated angiosuite for simulated EVAR for ruptured AAA. *Outcome* Feasibility of immersive angiosuite for emergency EVAR demonstrated. Specialists were faster than trainees. |
| **Anderson J *et al*. 2002** | Virtual reality training in interventional radiology: The Johns Hopkins and Kent Ridge digital laboratory experience | Training and simulation | Concept design and feasibility study | N/A | N/A | Design of a computer-based mixed reality training module for high-fidelity simulation ofinterventional procedures. *Outcome* Positive feedback from unspecified number of peripheral and neurological interventionalists |
