# Supplementary material for: Augmented Reality in Vascular and Endovascular Surgery: Scoping Review
Source: JMIR Serious Games. 2022 Sep 23;10(3):e34501. doi: 10.2196/34501 (PMC9547335; doi:10.2196/34501)
Supplement: Multimedia Appendix 2 [file games_v10i3e34501_app2.docx]

**Multimedia Appendix 2.** Imaging, registration, and display types within review.

| ***Author*** | ***Title*** | ***Image Type*** | ***Registration/ Tracking Type*** | ***Type of display*** |
| --- | --- | --- | --- | --- |
| **Swerdlow N.J *et al*. 2019** | Three-dimensional image fusion is associated with lower radiation exposure and shorter time to carotid cannulation during carotid artery stenting | 3D models from CTA/ MRA with superimposed CBCT | ‘*VesselNavigator*’ computer-based image fusion | Monitor |
| **Rynio P *et al*. 2019** | Holographically-Guided Endovascular Aneurysm Repair | Pre-operative CTA onto 2D fluoroscopy | Optical Simultaneous localisation and mapping (SLAM) (CarnaLife Holo) | AR HMD (Hololens) |
| **Grinshpoon A *et al. 2018*** | Hands-free augmented reality for vascular interventions | 3D model of heart | Human pose estimation | AR HMD (Hololens) |
| **Kilian-Meneghin J *et al*. 2018** | Evaluation of Methods of Displaying the Real-Time Scattered Radiation Distribution during Fluoroscopically-Guided Interventions for Staff Dose Reduction | Computer generated scatter beams | Microsoft Kinect (RGB and Depth) optical tracking | Monitor |
| **Schulz CJ *et al*. 2016** | Fusion Imaging to Support Endovascular Aneurysm Repair Using 3D-3D Registration | Pre-operative CTA registered with non-contrast Cone Beam CT (nCBCT) | Fusion image overlay software (3D-3D) | Monitor |
| **Cheng I *et al. 2014*** | An augmented reality framework for optimization of computer assisted navigation in endovascular surgery | Live ultrasound registered on 3D model derived from pre-scanned CT images | Electromagnetic (EM) and GPS tracking | Monitor |
| **Parrini S *et al. 2014*** | Augmented reality system for freehand guide of magnetic endovascular devices | 3D US derived model | Marker tracking (fiducial) | AR HMD (Integrated by research team. HMD by nVisor SX, NVIS Inc.) |
| **Van Den Berg J.C. 2013** | Three-dimensional image overlay to assist endovascular procedures | 3D image overlay using rotational XR/ CBCT | ‘*VesselNavigator*’  computer-based image fusion | Monitor |
| **Wang J *et al. 2012*** | Augmented reality during angiography: Integration of a virtual mirror for improved 2D/3D visualization | CT angiography (CTA) derived models used to derive 2D ‘mirror’ overlaid onto 2D fluoroscopic images | Fusion image overlay software (2D-3D) | Monitor |
| **Groher M *et al*. 2009** | Deformable 2D-3D registration of vascular structures in a one view scenario | CBCT and preoperative CTA | Fusion image overlay software (2D-3D) | Monitor |
| **Kaladji *et al*. 2019** | Fusion Imaging for EVAR with Mobile C-arm | Pre-operative CTA | Intensity based registration image overlay software (2D-3D) | Monitor |
| **Haxthausen F.V *et al*. 2019** | Catheter pose-dependent virtual angioscopy images visualized on augmented reality glasses | CTA derived 3D model | Feature-based registration with electromagnetic tracking | AR HMD- Hololens |
| **García-Vázquez *et al*. 2018** | Navigation and visualisation with HoloLens in endovascular aortic repair | 3D models derived from 3D Ultrasound and CTA | Feature-based registration with electromagnetic tracking | AR HMD  (Hololens) |
| **Koutouzi *et al*. 2017** | 3D Image Fusion to Localise Intercostal Arteries During TEVAR | CBCT and preoperative CTA fused onto 2D fluoroscopy | Intensity based registration with image overlay software (2D-3D) | Monitor |
| **Dumenil A *et al*. 2016** | A versatile intensity-based 3D/2D rigid registration compatible with mobile C-arm for endovascular treatment of abdominal aortic aneurysm | Preoperative CTA onto fluoroscopy | Intensity-based image overlay software (2D-3D) | Monitor |
| **Chaoyang Shi *et al. 2016*** | In vitro three-dimensional aortic vasculature modelling based on sensor fusion between intravascular ultrasound and magnetic tracker | Intravascular ultrasound derived 3D model | Electromagnetic tracking with hybrid motion sensor | Monitor |
| **Rolls A.E *et al*. 2016** | A Comparison of Accuracy of Image- versus Hardware-based Tracking Technologies in 3D Fusion in Aortic Endografting | CTA and CBCT derived 3D model fusion with 2D fluoroscopy | Image based vs Hardware based tracking | Monitor |
| **Koutouzi *et al*. 2015** | EVAR Guided by 3D Image Fusion and CO2 DSA: A New Imaging Combination for Patients With Renal Insufficiency | nCBCT fused with preoperative CTA and overlaid onto fluoroscopy | Intensity-based registration  image overlay software (3D-3D) | Monitor |
| **Dumenil A *et al*. 2015** | Safety and accuracy of endovascular aneurysm repair without pre-operative and intra-operative contrast agent | Unenhanced CT derived 3D model fused with CBCT | Intensity based registration with  image overlay software (3D-3D) | Monitor |
| **Fukuda T *et al*. 2013** | Evaluation of automated 2D-3D image overlay system utilizing subtraction of bone marrow image for EVAR: Feasibility study | CTA and CBCT derived 3D model with 2D fluoroscopy | Feature based registration image overlay software (2D-3D) | Monitor |
| **Carrell *et al*. 2010** | Feasibility and limitations of an automated 2D-3D rigid image registration system for complex endovascular aortic procedures | Pre-operative CTA onto fluoroscopy | Intensity-based registration image overlay software (2D-3D) | Monitor |
| **Lu W *et al*. 2020** | Augmented reality navigation to assist retrograde peroneal access for the endovascular treatment of critical limb ischemia | Preoperative CTA | Manual tracking by lining up markers on patients and glasses | AR HMD (Xiamen Minwei Ltd.) |
| **Goudeketting *et al*. 2018** | The use of 3D image fusion for percutaneous transluminal angioplasty and stenting of iliac artery obstructions: validation of the technique and systematic review of literature | Contrast enhanced MRA (CE MRA) registered onto CBCT | Intensity based registration image overlay software (3D-3D) | Monitor |
| **Schwein *et al*. 2017** | Magnetic resonance venography and three-dimensional image fusion guidance provide a novel paradigm for endovascular recanalization of chronic central venous occlusion | MRV and CBCT registered to each other. Overlaid onto 2D fluoroscopy | Intensity-based registration image overlay software (2D-3D) | Monitor |
| **Pietrabissa A *et al*. 2009** | Mixed reality for robotic treatment of a splenic artery aneurysm | CTA derived 3D model | Infrared optical tracking | AR HMD (Not specified) |
| **Mochizuki *et al. 2016*** | New simple image overlay system using a tablet PC for pinpoint identification of the appropriate site for anastomosis in peripheral arterial reconstruction | Preoperative CTA | Fiducial marker landmark-based registration and optical (RBG) tracking | Tablet PC |
| **Aly, Omar 2020** | Assisting Vascular Surgery with Smartphone Augmented Reality | Preoperative CTA to derive 3D model | Optical (RBG) augmented with gyroscopic tracking | Mobile phone |
| **Mangina E *et al*. 2018** | 3D modelling for augmented reality systems in novel vascular models | Preoperative CTA to derive 3D Model | N/A | N/A |
| **Burke E *et al*. 2017** | Augmented reality EVAR training in mixed reality educational space | 3D model derived from CTA and 3D printed model | Optical fucidial marker tracking | Mobile phone |
| **Bartesaghi S *et al*. 2017** | Spatial augmented reality and simulations to improve abdominal aortic aneurysm diagnosis and monitoring | CTA derived model | Optical fucidial marker tracking (ARToolkit) | Projector |
| **Rudarakanchana N *et al*. 2014** | Endovascular repair of ruptured abdominal aortic aneurysm: Technical and team training in an immersive virtual reality environment | VR rAAA from CTA derived models | ORCAMP simulated angio suite | Monitor |
| **Anderson J *et al*. 2002** | Virtual reality training in interventional radiology: The Johns Hopkins and Kent Ridge digital laboratory experience | 3D models of blood vessels | Simulated user interface with real guidewires- (hardware tracking) | Monitor |
